# Neurotrophin-Induced Migration and Neuronal Differentiation of Multipotent Astrocytic Stem Cells *In Vitro*

**DOI:** 10.1371/journal.pone.0051706

**Published:** 2012-12-12

**Authors:** Martha Douglas-Escobar, Candace Rossignol, Dennis Steindler, Tong Zheng, Michael D. Weiss

**Affiliations:** 1 Department of Pediatrics, University of Florida, Gainesville, Florida, United States of America; 2 Department of Neuroscience, University of Florida, Gainesville, Florida, United States of America; 3 McKnight Brain Institute, University of Florida, Gainesville, Florida, United States of America; University of California, San Diego, United States of America

## Abstract

Hypoxic ischemic encephalopathy (HIE) affects 2–3 per 1000 full-term neonates. Up to 75% of newborns with severe HIE die or have severe neurological handicaps. Stem cell therapy offers the potential to replace HIE-damaged cells and enhances the autoregeneration process. Our laboratory implanted Multipotent Astrocytic Stem Cells (MASCs) into a neonatal rat model of hypoxia-ischemia (HI) and demonstrated that MASCs move to areas of injury in the cortex and hippocampus. However, only a small proportion of the implanted MASCs differentiated into neurons. MASCs injected into control pups did not move into the cortex or differentiate into neurons. We do not know the mechanism by which the MASCs moved from the site of injection to the injured cortex. We found neurotrophins present after the hypoxic-ischemic milieu and hypothesized that neurotrophins could enhance the migration and differentiation of MASCs. Using a Boyden chamber device, we demonstrated that neurotrophins potentiate the *in vitro* migration of stem cells. NGF, GDNF, BDNF and NT-3 increased stem cell migration when compared to a chemokinesis control. Also, MASCs had increased differentiation toward neuronal phenotypes when these neurotrophins were added to MASC culture tissue. Due to this finding, we believed neurotrophins could guide migration and differentiation of stem cell transplants after brain injury.

## Introduction

Hypoxic ischemic encephalopathy (HIE) is the brain manifestation of systemic asphyxia [1]. HIE is a leading cause of neonatal mortality worldwide [2], [3] and affects 2–3 per 1000 newborns in the United States [4]. Supportive management of newborns with HIE includes maintenance of cerebral perfusion and adequate energy levels [5], [6]. With supportive care, 75% of newborns with severe asphyxia die or have permanent devastating neurological handicaps [7], [8]. Novel therapies to reduce brain cell apoptosis and necrosis have had very limited success [6], [9]–[12]. Only hypothermia [5], [13]–[15] and erythropoietin therapy have improved the morbidity and mortality rates of newborns with moderate to severe HIE [16]. Due to the poor outcomes associated with moderate and severe HIE and the substantial progress in stem cell biology, researchers have explored pioneer regenerative therapies. Regenerative therapies replenish lost cells by either endogenous neural progenitor cell (NPC) activation or by stem cell transplantation.

NPCs are tissue-specific undifferentiated cells that are able to self-renew and give rise to multi-lineage brain cells such as neurons, astrocytes, and oligodendrocytes [17]. In neonates, the subventricular zone (SVZ) contains multipotent NPCs that have the potential to replace damaged brain cells. Specifically, cells on the medial aspect of the SVZ tolerate the hypoxic-ischemic insult and help in the autoregeneration process [18]. Nevertheless, severe hypoxia-ischemia (HI) leads to a less-cellular SVZ [19], [20], suggesting that an exogenous source of stem cells may be necessary in severe HI.

Since endogenous repair is not always sufficient in severe HI, our laboratory used a regenerative approach to demonstrate that donor-derived multipotent astrocytic stem cells (MASCs) implanted into the cortex of a neonatal rat HI model moved to the area of injury and differentiated into neurons and mature glia [21]. Injected stem cells were located within the large ischemic areas of the injured hemisphere. Only a small subpopulation of MASCs in the injured cortex differentiated into neurons. No one knows the factors that promote the movement of the MASCs from the injection site towards the ischemic area and induce differentiation.

Neurotrophins are important cues for the migration and differentiation of neural stem cells [22], [23]. Neurotrophins are a family of growth factors that act through tyrosine kinase receptors and regulate the development and maintenance of brain cells by affecting neuronal survival, synaptogenesis, and brain plasticity [24]. The first neurotrophin discovered was neuronal growth factor (NGF). Further work identified other members of the family such as Glial Derived Neurotrophic Factor (GDNF), Brain Derived Neurotrophic Factor (BDNF), and Neurotrophin-3 (NT-3). Degenerative brain disease pathophysiology is related to abnormally low neurotrophin concentrations, and clinical improvements of such diseases correlate with increased neurotrophin concentrations [25] [1], [26]. In the neonatal period, neurotrophins and their receptors are essential for brain development. After brain insults, neurotrophins increase in number, suggesting that they have an endogenous protective mechanism that limits neuronal cell death [27]. Therefore, neurotrophins could mediate the migration of transplanted MASCs and once at the site of injury, enhance neuronal differentiation.

We hypothesized that neurotrophins present after the hypoxic-ischemic injury affect the migration and differentiation of MASCs. The primary goal of our study was to carry out *in vitro* experiments that explored whether various neurotrophins (GDNF, BDNF, NGF and NT-3) affected the migration and differentiation of MASCs. Our secondary goal was to determine the most effective trophic factor and the concentration needed to increase MASC migration and differentiation.

## Materials and Methods

### Preparation of MASCs for Culture

Protocols were in accordance with the International Guiding Principles for Animal Research and were approved by the University of Florida Institutional Animal Care and Use Committee. MASCs used for migration were derived from green fluorescent protein (GFP) transgenic mice (STOCK Tg GFPU 5Nagy/J, Stock # 03115, The Jackson Laboratory, Bar Harbor, ME). Newborn mice (P4–P5) were deeply anesthetized and decapitated. Subependymal zone tissue was obtained by brain micro-dissection. Tissue chunks were minced, incubated in 0.25% trypsin/EDTA (Atlanta Biologicals, Lawrenceville, GA) and dissociated into a single cell suspension. Single cells were pelleted and washed several times in medium before being plated in culture flasks with Dulbecco’s Modified Eagle Medium/F-12 (D-MEM/F12, 1:1, Gibco®, Grand Island, NY) and supplemented with 5% fetal bovine serum (FBS), epidermal growth factor (EGF, 20 ng/mL), and bovine fibroblast growth factor (bFGF, 10 ng/mL). The medium was changed every three days until the cells were confluent. MASC cultures (Figure S1A. MASC in culture) were generated as previously described [28]. These cultures yielded monolayers with the following expression rates: 95–100% astrocyte markers, 0% neuronal markers, and a small percentage of non-neurogenic microglia [21]. After two passages, confluent monolayers were collected via trypsinization and counted with a hemocytometer.

### Migration Assays

Although several methods are available to evaluate cell migration, the most widely accepted method is the Boyden chamber assay. Therefore, we chose to utilize this assay in our migration experiments. Migration was evaluated using a Boyden chamber CHEMICON® QCM™ 24-well Cell Migration Assay kit (ECM508, Millipore, Billerica, MA). This system consists of an upper chamber, a lower chamber, a polycarbonate membrane with 8 µm pores, and a thin layer of extracellular matrix separating the two chambers. The upper chamber was loaded with 50 µL MASCs (50×10^4^ MASCs). The lower chamber was loaded with the following concentrations of neurotrophins: recombinant human BDNF (Cat. #248-BD-025, R&D Systems, Inc®, Minneapolis, MN) at 10, 50, and 100 ng/mL, GDNF recombinant rat GDNF (Cat. # 512-GF-010, R&D Systems, Inc®) at 10, 50, and 100 ng/mL, recombinant mouse beta-NGF (Cat. #1156-NG-100, R&D Systems, Inc®) at 200, 300, and 400 ng/mL and recombinant human NT-3 (Cat. #267-N3-005, R&D Systems, Inc®) at 50, 100, and 150 ng/mL (Figure S2. Boyden Chamber). We chose the doses of neurotrophins after a pilot experiment in which the concentrations of the listed growth factors were analyzed 24 and 72 hours after HI injury (data not shown). The first two concentrations of each factor matched the average range of the concentrations found at 24 and 72 hours. The third concentration was twice as high as the highest average physiologic concentration.

After an incubation time of 24 and 72 hours at 37°C in humidified air with 5% CO_2_, the membrane was removed and the upper surface of the filter was scraped with a rubber scraper. The cells that migrated through the pores and adhered to the underside of the membrane were stained with cresyl violet (0.09%) and counted as per the manufacturer’s protocol (Figure S1B. MASC migration). The experiments were run in triplicate.

Each migration assay plate had positive and negative controls. *In vitro* studies of mesenchymal stem cell migration have demonstrated that medium supplemented with FBS stimulates the movement of the mesenchymal cells. Therefore, FBS served as a positive control on each plate to ensure that the cells seeded on the plate possessed the ability to migrate under the culture conditions. We used media supplemented with 10% FBS as a positive control and serum-free media as a true negative control. To distinguish between directed migration towards a chemoattractant and generalized random movement of stem cells (chemokinesis), we added a second, more stringent, control for chemokinesis. Stem cells were exposed to the same concentration of neurotrophin in the lower and upper chambers. Therefore, cell movement towards the bottom chamber was the result of chemokinesis, not migratory movement cued by differential concentrations of the neurotrophin.

ImageJ (NIH, Bethesda, MD) was used to count cells. Five random fields in each of three replicas of specific neurotrophin concentration were observed, resulting in 15 observations per condition. Migrated cells were defined as the number of cells per field and were compared with the controls.

### Differentiation Experiments

MASCs were grown as described above. After passage three, they were seeded at a density of 2×10^4^ MASCs per 1 mL on glass coverslips placed at the bottom of each well in a 24-well plate. To allow differentiation, coverslips were pre-coated with both Polyornithine (10 µg/mL, Sigma, St. Louis, MO) and Laminin (5 µg/mL, Sigma). MASCs were fed by renewing the medium (D-MEM/F12 without FGF and EGF) three times per week to allow differentiation. Each plate contained a negative control consisting of MASCs not exposed to any added differentiation factors. Extracellular matrix proteins such as Laminin and Polyornithine could play a role in the differentiation of stem cells to particular phenotypes; therefore, we used Laminin and Polyornithine coated glass coverslips on our negative controls to control for this possible effect. The factors and concentrations tested were BDNF (10, 50 and 100 ng/mL), GDNF (10, 50, and 100 ng/mL), NFG (200, 300, and 400 ng/mL) and NT-3 (50, 100, and 150 ng/mL). We assessed cell viability with a Trypan blue assay at 24 hours to determine whether higher concentrations of neurotrophins were toxic to the cells.

### Immunocytochemistry

After five days of differentiation, the immunocytochemistry was performed. We analyzed at five days because this time frame matched the relative time frame of our *in vivo* transplant experiments. A sample of cells from each condition was reserved for Trypan blue assay. This assay explored possible toxic effects of neurotrophins. The cells were fixed with cold 4% paraformaldehyde for 15 minutes, permeabilized, and blocked for 30 minutes at room temperature in PBS supplemented with 0.01 triton X-100, 10% FBS and 5% goat serum on the coverslips in the wells. After the blocking, the cells were incubated overnight with a primary antibody. Next, the cells were washed with PBS three times and incubated in the dark at room temperature for 30 minutes with appropriate secondary antibodies conjugated to various fluoresceins. The cells were coverslipped with Vectashield-mounting medium containing DAPI (Vector Laboratories, Inc, Burlingame, CA) and examined under a fluorescence microscope. The cells, which were β-3-tubulin positive, were counted in 15 high-power fields randomly chosen from each of the groups. The primary antibody was mouse anti-β-tubulin III (1: 500, Promega Corporation, Madison, WI) and was used to detect immature neurons. The secondary antibody was Cy3 (1:300, The Jackson Laboratory, Bar Harbor, ME).

Photographs of immunostained cells were taken utilizing a high-resolution CCD camera (Nikon, Tokyo, Japan) attached to a Leica microscope. ImageJ (NIH) was used to count GFAP and β-3 tubulin positive cells in 15 observations per condition (3 replicas per each concentration of specific neurotrophin and 5 random fields per replica).

### Statistical Analysis

Data analysis was performed using PRISM® (GraphPad Software, San Diego, CA). All means are presented as +/− the standard error of the mean (SEM). Differences in quantitative variables (cells migrated or differentiated) between groups and control were tested with one-way ANOVA with *post hoc* Bonferroni method to correct for multiple comparisons. For all differentiation experiments, the person counting cells was blinded to the group’s identities. The level of significance was set at P<0.05.

## Results

### Migration Assay Results

**Figure 1:**
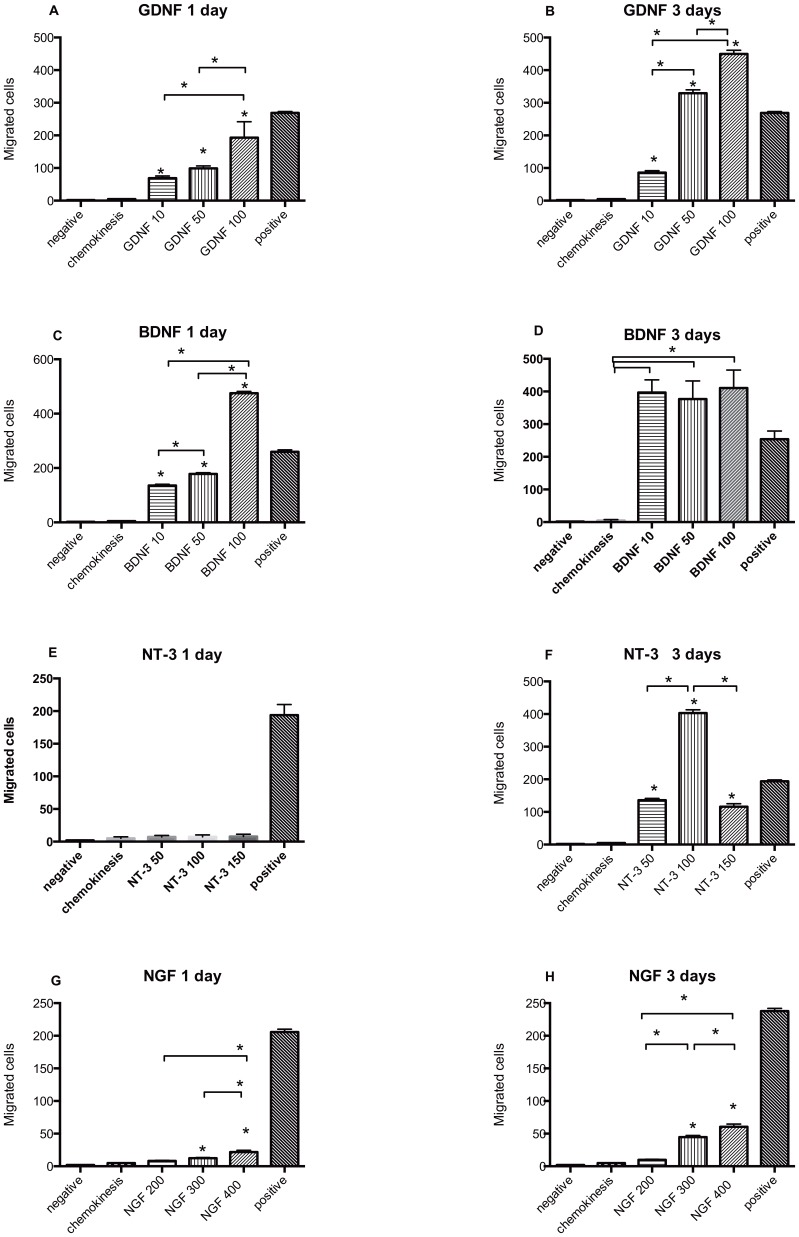
Migrated MASC (Mean and SEM) under different conditions (y axis). Panel A and B: GDNF at 10, 50 and 100 ng/mL concentrations during 1 and 3 days, respectively. Panel C and D: BDNF at 10, 50 and 100 ng/mL concentrations during 1 and 3 days. Panel E and F: NT-3 at 50, 100 and 150 ng/mL during 1 and 3 days, respectively. Panel F and G: NGF at 200, 300 and 400 ng/mL during 1 and 3 days, respectively. Each panel includes negative control (no neurotrophins added), chemokinesis (random movement control) and positive control (10% FBS). Statistically significant differences between chemokinesis and specific concentrations of neurotrophin were noted with an asterisk (p<0.05). The bars over the groups show statistically significant differences between different neurotrophins’ concentrations.

The mean number of migrated cells showed a dose response to GDNF concentration at days one and three (Table S1). On day one, the mean number of migrated cells was significantly greater than the negative control at GDNF concentrations of 10 ng/mL, 50 ng/mL, and 100 ng/mL, p<0.05 (Figure 1A) but less than the positive control. Similar results occurred on day three (Figure 1B) and the mean number of migrated cells with GDNF at 50 and 100 ng/mL was 1.7 times higher than the positive control. Day 3 had up to a 3-fold increase in migration when compared to day 1. Overall, GDNF produced a 14- to 92-fold increase in migration over chemokinesis.

MASCs had a response to BDNF exposure similar to GDNF exposure. On day one, the mean number of migrated cells was significantly greater than the chemokinesis controls at BDNF concentrations of 10, 50, and 100 ng/mL, p<0.05 (Figure 1C). Migration of cells induced by BDNF at 100 ng/mL (one-day exposure) was 1.8 times higher than the positive control. BDNF induced a 56- to 97-fold increase in migration over chemokinesis at 24 h. After three days of BDNF exposure migrated cells were 1.6 times higher than the positive control (Figure 1D).

After one day of NT-3 exposure, the cell migration response was not different from the negative control (Figure 1 E). However, after three days of exposure to NT-3, the mean number of migrated cells was significantly greater than the control, p<0.05 (Figure 1F). Exposure to NT-3 for 3 days induced a 24- to 84-fold increase in migration when compared to controls exposed to chemokinesis. We observed a greater migration in response to NT-3 at 100 ng/mL than at 50 ng/mL but a decrease at 150 ng/mL. After 3 days exposure to NT-3 at 100 ng/mL, migrated cells were 2 times higher than the positive control (Figure 1F).

After one day of NGF exposure, the mean number of exposed migration cells was significantly greater than the cells exposed to chemokinesis, p<0.05 (Figure 1G) but only 10% of the positive control. Migration increased up to 3-fold from day 1 to day 3. NGF produced a modest response compared to the other neurotrophins tested. Exposure to NGF for 3 days caused a 1.9- to 12.4-fold increase in cell migration over chemokinesis, p<0.05 (Figure 1H) but only 1/4 of the migration obtained with the positive control.

### Differentiation Experiment Results

**Figure 2:**
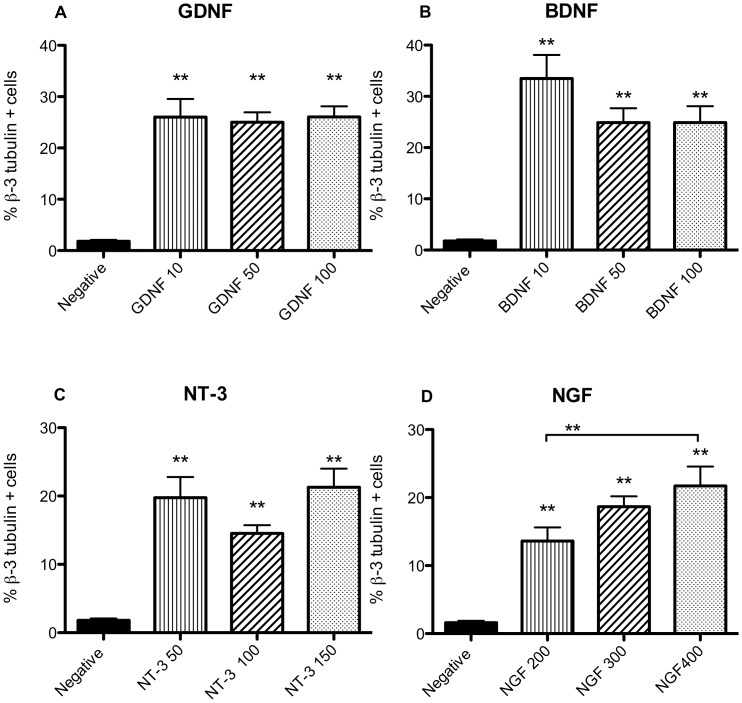
Percentage β-3 tubulin positive cells (Mean and SEM) under different neurotrophins conditions. Panel A: GDNF at 10, 50 and 100 ng/mL concentrations. Panel B: BDNF at 10, 50 and 100 ng/mL concentrations. Panel C: NT-3 at 50, 100 and 150 ng/mL. Panel D: NGF at 200, 300 and 400 ng/mL. The asterisks (**) represent statistically significant differences between the negative control and specific neurotrophin (p<0.01). The only significant difference between different concentrations of neurotrophins existed between NGF 200 and 400 ng/mL.

Trypan blue assays at 24 and 72 hours demonstrated that 97–98% of cells under all conditions survived. GDNF, BDNF, NT-3, and NGF increased cell differentiation into a neuronal phenotype when compared with negative controls, but only NGF showed an incremental dose response (Table S2). Cells had a flat response to GDNF doses of 10, 50 and 100 ng/mL. However, significantly more cells were β-3 tubulin positive than the negative control, p<0.01 (Figure 2A). Overall BDNF increased differentiation of cells at doses of 10, 50 and 100 ng/mL when compared with the negative control, p<0.01 (Figure 2B). NT-3 exposure also produced an increased percentage of β-3 tubulin positive cells at doses of 50, 100 and 150 ng/mL when compared with negative controls, p<0.01 (Figure 2C). Cells subjected to NGF showed an escalating dose-response of β-3 tubulin positive cells at doses of 200, 300 and 400 ng/mL that was significantly different than the negative control, p<0.01 (Figure 2D).

## Discussion

The first critical step in stem cell engagement during regeneration is migration toward injured areas of the brain. Thus, migration plays a central role in brain repair. Our previous work demonstrated that MASCs moved towards the region of injury following HI by an unknown homing mechanism. Migration of neuronal progenitor cells towards injured tissue is a multistep process mediated by a series of sequential cellular interactions in which the generation of chemotaxic gradients plays a key role [29]. The signals that induce migration may be generated locally as components of the extracellular matrix or released as soluble substances such as chemokines and growth factors. Although several methods are available to evaluate cell migration, the most widely accepted method is the Boyden Chamber assay. Therefore, we elected to utilize this assay in our migration experiments. Using this system, we demonstrated that neurotrophins present in the post-injury milieu (unpublished data) increase the migration of MASCs *in vitro*. We examined migration at two time points. Our rationale for this approach is that most transplanted cells migrate at a rate of 100 µm/hour. Therefore, most of the stem cells reach the injured lesion in 24–72 hours. More important, a stem cell transplant three days after HI likely provides the most cell engraftment, survival, and differentiation [30]. Therefore, we chose an initial time point of 24 hours and a later time point of 72 hours to quantify migration. Our results demonstrate that GDNF and BDNF at physiologic concentrations produce the greatest movement of cells at 24 hours. Since both GDNF and BDNF are present at physiologic concentrations after injury, both factors may help mediate the movement of the MASCs toward the site of injury *in vivo*. Our migration data at 24 hours reveals that the number of cells is enhanced 2-fold and 3-fold by doubling the physiologic levels of GDNF and BDNF, respectively. These neurotrophins could supplement the endogenous milieu to enhance the movement of the transplanted MASCs to the site of injury.

Adult stroke also shows factor-mediated movement. For example, GDNF increased migration and differentiation in animal stroke models [31], [32]. Furthermore, adult NPCs that were genetically modified to secrete more GDNF and then transplanted after ischemic insult had increased migration and neuronal phenotype differentiation when compared with controls (naïve NPCs) in *in vivo* studies [33]. Moreover, these grafted neural stem cells modified by the GDNF gene had higher survival rates and differentiation after stroke, less apoptosis, and greater expression of BDNF and NT-3 [31].

In the second portion of our migration experiments, we examined the possibility of increased migration over time. We selected a period of 3 days because at the migration rate of 100 µm/hour most of our transplanted MASCs in our previous *in vivo* study should have reached the area of injury. Prolonged exposure to GDNF at physiologic and supra-physiologic concentrations increased migration after injury. Interestingly, prolonged exposure to BDNF did not result in an increase in migration. However, exposure to NGF at a physiologic concentration for 3 days increased migration. These data demonstrate that an additional 48-hour exposure to factors can enhance migration. Multiple doses over several days might increase the movement of the transplanted cells from the transplant site to the injured tissue.

Neurons are particularly sensitive to hypoxic-ischemic injury. Following injury, one of the goals of neuroregeneration is to restore neuronal and astrocyte populations. MASCs are good candidates to restore the injured brain since they are derived from the SEZ and are programmed to migrate to various brain regions. In our previous work, we demonstrated that this migration occurs after injury and some cells differentiate into neurons. We need a basic understanding of the factors that may mediate the differentiation process in order to understand the factors that produce neuronal differentiation. With this knowledge, we may be able to enhance neuronal differentiation.

We began to explore this mechanism in our experiments by examining candidate neurotrophins at concentrations found in the post-injury milieu. Our results demonstrate that GDNF and BDNF at physiologic concentrations present post hypoxia-ischemia have the greatest impact in increasing neuronal differentiation. However, we did not find a further increase in neurons at concentrations above the physiologic level. Prior *in vitro* experiments demonstrated that GDNF is a potent chemoattractant and chemo-kinetic factor for neural precursor cells [34]. The present study showed that GDNF is a potent stimulus for the migration and differentiation of MASCs. Other *in vitro* studies demonstrated this finding [35] and showed that adding GDNF (50 ng/mL) to cultured NPCs (derived from the medial ganglionic eminence) for 48 hours increased cell differentiation towards GABA-positive cells and enhanced the cells’ neuronal morphology and motility. GDNF may interact with or affect the expression of other neurotrophins, adding exponentially increased neuroprotective effects.

Similar evidence exists for BDNF as GDNF. BDNF is an important factor during brain development [32] and provides a directional cue that promotes the migration of cerebellar granular cells [36]. We found evidence that BDNF concentrations are increased after neonatal HI (unpublished data) as well as in the post-stroke milieu [37]. Researchers have postulated that BDNF increases the migration of stem cells [38], [39]. Our data support the notion that BDNF potentiates stem cell migration even after a short exposure time (24 hours) when added to *in vitro* models. Most recently, Zhang et al. [40] published that BDNF (10 ng/mL) added to the culture media induced differentiation of umbilical cord mesenchymal stem cells towards a neuronal phenotype. Our results agree with the aforementioned and show that low doses of BDNF increase MASCs to differentiate toward a neuronal fate. Other studies have demonstrated that protoplasmic astrocytes in co-culture with NPCs that over-express BDNF induce NPC neuronal fate [41]. In this co-culture model, the media supernatant had a high content of BDNF, and the addition of anti-BDNF antibody to the medium reduced the number of neurons that differentiated from NPCs. In mouse SVZ explant cultures, a low dose of BDNF increased migration of NPCs, while high doses of BDNF increased differentiation [42], [43]. In contrast, in our experiments BDNF stimulated migration in a dose-dependent manner while differentiation increased independent of the dose.

In addition to BDNF, NGF and NT-3 also increased the number of cells that differentiated into neurons. NGF is important in promoting axonal regeneration after transplantation of NPCs in models of spinal injury [44]. When low doses of NGF are added to the culture medium of human embryonic stem cells, the percentage of cells that differentiate toward a neuronal phenotype doubles [45]. In our experiments, NGF increased migration and differentiation toward a neuronal phenotype of MASC. In contrast with our results, *in vitro* experiments have demonstrated that NGF improves Schwann precursor cell motility but does not affect migration [34]. One possible explanation is that different progenitor cells do not respond to neurotrophin in the same way. Based on this information from the literature and our current data, we found that MASCs exposed to NGF have a modest increase in migration and a dose-related increase in differentiation toward neuronal phenotype. NGF is the only tested neurotrophin that may increase the percentages of neuronal phenotypes when administered in supraphysiologic concentrations. As a predifferentiation strategy, cells exposed to the neurotrophin prior to transplant may increase the neuronal phenotypes pre-transplant.

NT-3 displays neuroprotective properties and when combined with NSCs transplanted in an HI rodent model improves neuronal differentiation and survival [46]. In our *in vitro* experiments, we found that even low doses of NT-3 increased migration in a non-linear curve response with an optimal dose of 100 ng/mL of NT-3. NT-3 also enhanced differentiation but did not show a dose response. Because the soluble form of NT-3 is bioactive for only a short time, other researchers have attempted to bind NT-3 to carriers [47]. When NT-3 is loaded with chitosan carriers, NPCs show enhanced differentiation toward neuronal phenotypes and increased survival [48]. Therefore, NT-3 (in its soluble or carrier-loaded form) increases differentiation of NPCs toward a neuronal phenotype. Our results are in agreement with other reports that have shown an increase in NPC migration when exposed to normally-expressed NT-3 or over-expressed NT-3 by lentivirus [49], [50].

Following HIE, a complicated cascade of pathophysiologic processes are unleashed including excitotoxicity, oxidative stress, inflammation, and cell death via necrosis and apoptosis. These processes can lead to long-term neurologic deficits [51]. The post-injury time course can be divided into a latent (0–6 hours), secondary (6–72 hours) and tertiary phase (>72 hours) [52]. During the tertiary phase, neurons and glial cells are lost due to chronic loss of trophic factors, loss of synaptic input from neighboring cells, and loss of or failure of recruitment of new progenitor neural stem cells and glial progenitor cells [53], [54].

At present, the goal of regenerative medicine involves providing tropic factors and NPCs to prevent the disruption of the endogenous neuronal networks [55]. The survival of transplanted NSCs was limited in these experiments for several potential reasons [56]–[58]. One of the reasons is that the environment may not be ideal for survival [52]. To date, studies have not enhanced the environment by combining the transplanted cells with survival factors. Several groups have attempted to transplant cells that secrete survival factors to improve endogenous tissue survival [46]. Our data suggest a combination of neurotrophins and MASCs may help the endogenous tissue and increase the movement of MASCs toward the site of injury. GDNF plays a crucial role in neuroprotection [12], [59] and is a chemoattractant factor for neuronal precursors in the rostral migratory stream [60], [61]. GDNF may interact or affect the expression of other neurotrophins, adding exponential neuroprotective effects.

We omitted a few considerations in our research, which we will address in future studies. We simplified our approach to evaluate migration and differentiation of NPCs *in vitro*, but we understand that *in vivo* models are more complex and frequently involve inflammatory responses. Various mechanisms could be responsible for the preferential movement of stem cells towards the area of brain injury [46], [62]. However, inflammation is common to many modes of brain injury [63], [64]. The addition of inflammatory mediators (such as interleukins) to the design may change the neurotrophins’ efficacy as homing factors and should be explored in the future. Another important consideration is the potential interaction between different neurotrophins. For example, *in vitro* experiments demonstrated that GDNF-induced branching of cultured ciliary neurons could be inhibited by BDNF [37]. Therefore, before researchers use neurotrophins together, they should test for potential interactions.

Most of the NPC migration assays reported in the literature only expose the NPCs to chemoattractants for 24 hours. However, because our laboratory is currently doing stem cells transplants 1 to 3 days after HI, we exposed the NPCs to chemoattractants for 1 and 3 days to examine their response to neurotrophins.

In the future neurotrophin receptor blockers should be used to further corroborate neurotrophin-induced migration and differentiation. We speculate that by incorporating the homing factors into the transplant paradigm, we may improve the migration and differentiation of NPC. Homing factors may also improve the localization of NPCs and thus improve NPCs incorporation into the local host circuitry.

We also recognize that the post-injury milieu is a very complicated environment with many other factors present, which may partially or synergistically affect the migration and neuronal differentiation of transplanted MASCs. Preconditioning cells with trophic factors before transplantation is a very attractive concept that could improve migration, differentiation, and survival. When deciding the timing of exposure of the cells to the neurotrophins, we noted that most reports on preconditioning of stem cells are done for 24 hours, but our laboratory is currently performing stem cell transplants up to 3 days after HI. Thus, we chose to expose the stem cells to neurotrophins for 1 and 3 full days. Our results show that neurotrophins at a small concentration exert effects very rapidly and can modify NPC fate and directional movement.

We speculate that in the future researchers will incorporate into their protocols controlled exposure to neurotrophins before cell transplant to enhance the cells’ migration profile and to direct their fate. Our next set of experiments should also explore whether neurotrophins could improve the integration of transplanted cells into the local host circuitry.

### Conclusion

Increasing migration and differentiation of stem cells transplanted in stroke and HI models of brain injury are a critical juncture for translational clinical applications. We found that the addition of GDNF, NGF, EGF, and NT-3 to *in vitro* cultures of MASCs increased migration and differentiation toward neuronal phenotypes. Our results indicate that MASCs exposed to one day of GDNF at 100 ng/mL and BDNF at 100 ng/mL have the greatest *in vitro* migration. In addition, MASCs exposed to three days of NT-3 at 100 ng/mL and NGF at 400 ng/mL have the most migration. Our results indicate that MASCs exposed to GDNF at10 ng/mL, BDNF at 10 ng/mL, NT-2 at 50 ng/mL and NGF at 400 ng/mL have the most differentiation toward neuronal phenotypes. We describe how even brief exposure (such as 24 hours) to neurotrophins can change the phenotype of NPCs. More studies are needed to determine whether the same response is obtained with other NPCs transplanted after brain injury.

## Supporting Information

Figure S1 **MAS culture and migration.** Panel S1A Microphotography of MASCs in culture. MASCs are derived from the SVZ of GFP transgenic newborn mice (post-natal day 2 – 4). Panel S1B. Microphotography of membrane with micro pores and migrated cell (from the migration experiments). Bar = 200 µm.
(TIF)

Figure S2 **Boyden chamber.** The Boyden upper chambers were loaded with 50×10^4^ stem cells and the lower chambers were loaded with different concentrations of neurotrophins. A polycarbonate membrane with pores and a thin layer of extracellular matrix were between the two chambers. Migrated cells were counted after 24 and 72 hours of migration.
(TIFF)

Table S1 **MASCs migration response to different neurotrophins.** Concentrations are presented in ng/mL and results are presented as mean number of migrated cells and its 95% confidence intervals.
(DOC)

Table S2 **Percentage β-3 tubulin positive cells after exposure to different neurotrophins.** Concentrations of neurotrophins are presented in ng/mL and results are presented as mean number of migrated cells and its 99% confidence intervals.
(DOC)
