# Supplementary figures and images for: Neurotrophin-Induced Migration and Neuronal Differentiation of Multipotent Astrocytic Stem Cells In Vitro
Source: PLoS One. 2012 Dec 12;7(12):e51706. doi: 10.1371/journal.pone.0051706 (PMC3520915; doi:10.1371/journal.pone.0051706)

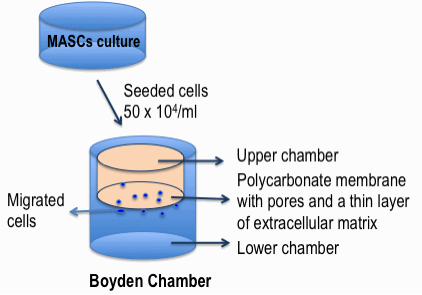

Supplement: Figure S2 — Boyden chamber. The Boyden upper chambers were loaded with 50×104 stem cells and the lower chambers were loaded with different concentrations of neurotrophins. A polycarbonate membrane with pores and a thin layer of extracellular matrix were between the two chambers. Migrated cells were counted after 24 and 72 hours of migration. (TIFF) [file pone.0051706.s002.tiff]
