# Supplementary material for: Neurotrophin-Induced Migration and Neuronal Differentiation of Multipotent Astrocytic Stem Cells In Vitro
Source: PLoS One. 2012 Dec 12;7(12):e51706. doi: 10.1371/journal.pone.0051706 (PMC3520915; doi:10.1371/journal.pone.0051706)
Supplement: Table S1 — MASCs migration response to different neurotrophins. Concentrations are presented in ng/mL and results are presented as mean number of migrated cells and its 95% confidence intervals. (DOC) [file pone.0051706.s003.doc]

**Table S1**. MASCs migration in response to neurotrophins

| **Neurotrophin**  **Concentration (ng/mL)** | | **Day 1** | | **Day 3** | |
| --- | --- | --- | --- | --- | --- |
|  |  | **# Migrated cells (mean)** | **95% CI** | **# Migrated cells (mean)** | **95% CI** |
| **Negative control** | N/A | 2.01 | 1.64, 2.36 | 3.04 | 1.97, 4.56 |
| **Chemokinesis control** | N/A | 4.87 | 3.55, 6.19 | 4.37 | 3.21, 6.98 |
| **GDNF** | 10 | 68.87 | 54.55, 83.18 | 86.13 | 74.21, 98.06 |
|  | 50 | 99.00 | 82.85, 115.10 | 329.40 | 307.30, 351.50 |
|  | 100 | 192.90 | 87.84, 298 | 449.71 | 425.40, 474.10 |
| **BDNF** | 10 | 135.50 | 125.50, 145.60 | 135.5 | 125.5, 145.6 |
|  | 50 | 178.30 | 169.30, 187.30 | 178.3 | 169.3, 187.3 |
|  | 100 | 475.10 | 460.80, 489.30 | 475.3 | 460.8, 489.3 |
| **NT-3** | 10 | 7.13 | 5.78, 8.49 | 136.2 | 125.4, 147 |
|  | 100 | 7.33 | 5.73, 8.94 | 403.1 | 381.3, 424.8 |
|  | 150 | 7.8 | 5.87, 9.73 | 116.1 | 96.75, 135.4 |
| **NGF** | 200 | 8.33 | 6.84, 9.82 | 9.6 | 7.41, 11.29 |
|  | 300 | 12.47 | 10.25, 14.69 | 44.7 | 39.19, 50.14 |
|  | 400 | 21.6 | 15.73, 27.47 | 60.53 | 51.42, 69.65 |
| **Positive control** | FBS 10% | 268.8 | 259.8, 277.8 | 281.5 | 260.3, 295.2 |

**Table S1**. MASCs response to different neurotrophins. Concentrations are presented in ng/mL and results are presented as mean number of migrated cells and its 95% confidence intervals.
