# Supplementary material for: Neurotrophin-Induced Migration and Neuronal Differentiation of Multipotent Astrocytic Stem Cells In Vitro
Source: PLoS One. 2012 Dec 12;7(12):e51706. doi: 10.1371/journal.pone.0051706 (PMC3520915; doi:10.1371/journal.pone.0051706)
Supplement: Table S2 — Percentage β-3 tubulin positive cells after exposure to different neurotrophins. Concentrations of neurotrophins are presented in ng/mL and results are presented as mean number of migrated cells and its 99% confidence intervals. (DOC) [file pone.0051706.s004.doc]

**Table S2**. Percentage β-3 tubulin positive cells under different neurotrophins conditions

| **Neurotrophin** | **Concentration (ng/mL)** | **β-3 tubulin (%) cells** | **SEM** | **99% CI** |
| --- | --- | --- | --- | --- |
| **Negative control** | N/A | 1.81 | 0.28 | 1.01, 2.63 |
| **GDNF** | 10 | 26.00 | 3.56 | 15.51, 36.49 |
|  | 50 | 25.00 | 1.94 | 19.29, 30.71 |
|  | 100 | 26.06 | 2.06 | 20.00, 32.13 |
| **BDNF** | 10 | 33.47 | 4.61 | 19.76, 47.18 |
|  | 50 | 24.87 | 2.83 | 16.44, 33.29 |
|  | 100 | 24.80 | 3.22 | 15.29, 34.44 |
| **NT-3** | 10 | 19.76 | 3.02 | 10.94, 28.59 |
|  | 100 | 14.53 | 1.22 | 10.97, 18.09 |
|  | 150 | 21.29 | 2.71 | 13.39, 29.20 |
| **NGF** | 200 | 13.61 | 8.47 | 7.83, 19.39 |
|  | 300 | 18.67 | 6.44 | 14.27, 23.06 |
|  | 400 | 21.72 | 12.06 | 13.48, 29.96 |

**Table S2**. Percentage β-3 tubulin positive cells after exposure to different neurotrophins. Concentrations of neurotrophins are presented in ng/mL and results are presented as mean number of migrated cells and its 99% confidence intervals
